# Mitosis Counting in Breast Cancer: Object-Level Interobserver Agreement and Comparison to an Automatic Method

**DOI:** 10.1371/journal.pone.0161286

**Published:** 2016-08-16

**Authors:** Mitko Veta, Paul J. van Diest, Mehdi Jiwa, Shaimaa Al-Janabi, Josien P. W. Pluim

**Affiliations:** 1 Medical Image Analysis Group (IMAG/e), Eindhoven University of Technology, Eindhoven, The Netherlands; 2 Department of Pathology, University Medical Center Utrecht, Utrecht, The Netherlands; 3 Department of Pathology, VU University Medical Center, Amsterdam, The Netherlands; 4 Image Sciences Institute, University Medical Center Utrecht, Utrecht, The Netherlands; Universita degli Studi di Torino, ITALY

## Abstract

**Background:**

Tumor proliferation speed, most commonly assessed by counting of mitotic figures in histological slide preparations, is an important biomarker for breast cancer. Although mitosis counting is routinely performed by pathologists, it is a tedious and subjective task with poor reproducibility, particularly among non-experts. Inter- and intraobserver reproducibility of mitosis counting can be improved when a strict protocol is defined and followed. Previous studies have examined only the agreement in terms of the mitotic count or the mitotic activity score. Studies of the observer agreement at the level of individual objects, which can provide more insight into the procedure, have not been performed thus far.

**Methods:**

The development of automatic mitosis detection methods has received large interest in recent years. Automatic image analysis is viewed as a solution for the problem of subjectivity of mitosis counting by pathologists. In this paper we describe the results from an interobserver agreement study between three human observers and an automatic method, and make two unique contributions. For the first time, we present an analysis of the object-level interobserver agreement on mitosis counting. Furthermore, we train an automatic mitosis detection method that is robust with respect to staining appearance variability and compare it with the performance of expert observers on an “external” dataset, i.e. on histopathology images that originate from pathology labs other than the pathology lab that provided the training data for the automatic method.

**Results:**

The object-level interobserver study revealed that pathologists often do not agree on individual objects, even if this is not reflected in the mitotic count. The disagreement is larger for objects from smaller size, which suggests that adding a size constraint in the mitosis counting protocol can improve reproducibility. The automatic mitosis detection method can perform mitosis counting in an unbiased way, with substantial agreement with human experts.

## Introduction

Tumor proliferation speed is an important biomarker for breast cancer. [1] The most common and accessible method for assessing tumor proliferation speed is by counting of mitotic figures in hematoxylin & eosin (H&E) stained histological slide preparations. The mitotic count, expressed as the mitotic activity index (MAI, number of mitoses in 2 mm^2^ tissue area), is part of the modified Bloom-Richardson grading system [2], but is also considered an independent prognostic factor. [3] Although mitosis counting is routinely performed in almost every pathology lab, the procedure is tedious, and can suffer from reproducibility problems (particularly between non-specialized pathologists) that are caused by the underlying subjectivity and difficulty of the task.

The appearance of mitotic figures in H&E stained preparations can in theory be summarized by a list of a few typical characteristics. Mitotic figures appear as hyperchromatic objects without nuclear membrane and hairy extensions of nuclear material. However, in practice, scanning the histological slides for objects exhibiting these characteristics is not trivial as there are many doubtful instances where subjective decisions must be made. This can in some cases lead to wrong estimation of the tumor proliferation speed and in turn to wrong indications for systemic therapy.

Inter- and intraobserver reproducibility of mitosis counting can be improved when a strict mitosis counting protocol is defined and followed. [4] Previous studies of the observer agreement on mitosis counting have examined only the agreement in terms of the mitotic count or the mitotic activity score. Studies of the observer agreement at the level of individual objects, which can provide more insight into how pathologists perform this task, have not been performed thus far. The main reason for this is the technical difficulty of performing such studies with conventional light microscopes and glass slides. With this equipment, it is not possible to precisely mark the locations of very small objects such as mitoses, which is necessary in order to assess the object-level agreement.

At present time, a fully digital pathology workflow, where traditional glass slides are replaced with digital slides that are viewed on a computer screen, is feasible and being implemented in pathology labs. In addition to many workflow benefits, digital slides offer the possibility for precise annotations and use of image analysis techniques to tackle some of the known drawbacks of manual analysis by pathologists such as the previously mentioned subjectivity issues.

The development of automatic mitosis detection methods has received large interest in recent years. [5–8] Automatic image analysis is viewed as a solution for the problem of subjectivity of mitosis counting by pathologists. One additional benefit of automatic analysis is that it can save pathologists valuable time by fully or partially automating this tedious task. Although state-of-the-art automatic mitosis detection methods approach the performance of human expert observers, this is achieved in relatively controlled conditions where automatic mitosis detectors are trained and evaluated on datasets originating from the same pathology lab. In a real world scenario, however, mitosis detection must be applied to digital slides originating from a variety of different pathology labs. Due to the variability of the tissue appearance between labs the performance of the methods can be sub-optimal when applied to “external” image data.

In this paper we describe the results from an interobserver agreement study between three human observers and an automatic method, and make two unique contributions towards improving the reproducibility of mitosis counting. For the first time, we present an analysis of the object-level interobserver agreement on mitosis counting. Furthermore, we train an automatic mitosis detection method that is robust with respect to staining appearance variability and compare it with the performance of expert observers on an “external” dataset, i.e. on histopathology images that originate from pathology labs other than the pathology lab that provided the training data for the automatic method.

## Materials and Methods

### Dataset

The analysis of the observer agreement and the evaluation of the automatic mitosis detection method is performed with the dataset previously used by Al-Janabi et al. to compare mitosis counting on conventional glass slides and digital slides. [9] The dataset consists of 100 consecutive breast cancer cases equally sourced from two pathology labs in the Netherlands. These include six core needle biopsies in cases undergoing neo-adjuvant chemotherapy and 94 resections. From each case, one representative slide routinely stained with H&E and containing tumor region was selected by two pathologists. The glass slides were scanned with a Leica SCN400 whole-slide image scanner at × 40 magnification and a spatial resolution of 0.25 μm/pixel. Within each slide, one tumor region of size 2 mm^2^ was marked for mitosis counting. The regions were selected based on the standard guidelines that state that mitosis counting should be performed in regions with high cellularity at the tumor periphery. Regions with tissue preparation artifacts were avoided. Mitosis counting on digital slides was performed independently by three pathologists following a strict scoring protocol using the Digital Image Hub software from Leica. For the majority of cases, the locations of the counted mitotic figures were recorded. Eighty-four cases have annotations of the locations of the mitotic figures by all three observers. The ground truth mitotic figure annotations are provided as pixel coordinates centered at the mitotic figure.

### Automatic mitosis detection

The automatic mitosis detection method was trained with the dataset from the AMIDA13 challenge. [5] This dataset consists of 23 breast cancer cases from the Department of Pathology at the University Medical Center, Utrecht, the Netherlands. Each case is represented by one routinely stained H&E slide and one representative tumor region within the slide, both selected by an expert pathologist. The slides were digitized with an Aperio XT scanner at ×40 magnification and a spatial resolution of 0.25 μm/pixel. Within each region, mitotic figures were annotated based on the consensus of two observers. The dataset is split into two subsets: a subset of 12 cases with 550 annotated mitotic figures used for training the automatic mitosis detection method and a subset of 11 cases with 533 annotated mitotic figures used for validation. A more detailed description of the dataset can be found in the overview paper of the challenge [5] and on the AMIDA13 challenge website (http://amida13.isi.uu.nl) where the data is available for download.

Mitotic figures were detected with an automatic image analysis method based on deep convolutional neural networks. These methods are part of the field of deep learning that has recently significantly improved the state of the art in many machine learning and computer vision tasks. [10] This success is largely owed to their ability to learn a hierarchical set of highly discriminative features directly from the image data.

The neural network architecture that was used for the mitosis detection in this paper is similar to the best-performing method of the AMIDA13 challenge. [5,6] The automatic method was trained with the 12 cases from the AMIDA13 training subset. The optimal operating point for the detection method was selected based on the 11 cases in the validation subset using the Dice similarity coefficient (see the following subsection) as the optimization criterion. The trained detector was then used to automatically detect mitotic figures, and compute the mitotic count and score in the independent dataset of 100 cases. We note here that these 100 cases originate from pathology labs that are different from the pathology lab that provided the images for the AMIDA13 dataset, and in addition, they were digitized with different scanning equipment.

Since the AMIDA13 data originates from a single pathology lab it does not account for inter-lab staining variations. In order to improve the generalization of the detection method to datasets from other pathology labs, a staining normalization procedure [11,12] was applied to the image data prior to the training of the deep convolutional neural network model. The same procedure is also applied to the test images prior to the detection. Additionally, during the training process, the training samples were augmented by creating new samples by random perturbation of the color and contrast of the original samples. This procedure has a regularization effect and makes the method more robust to such variations, which are not completely eliminated by the staining normalization.

**Fig 1:**
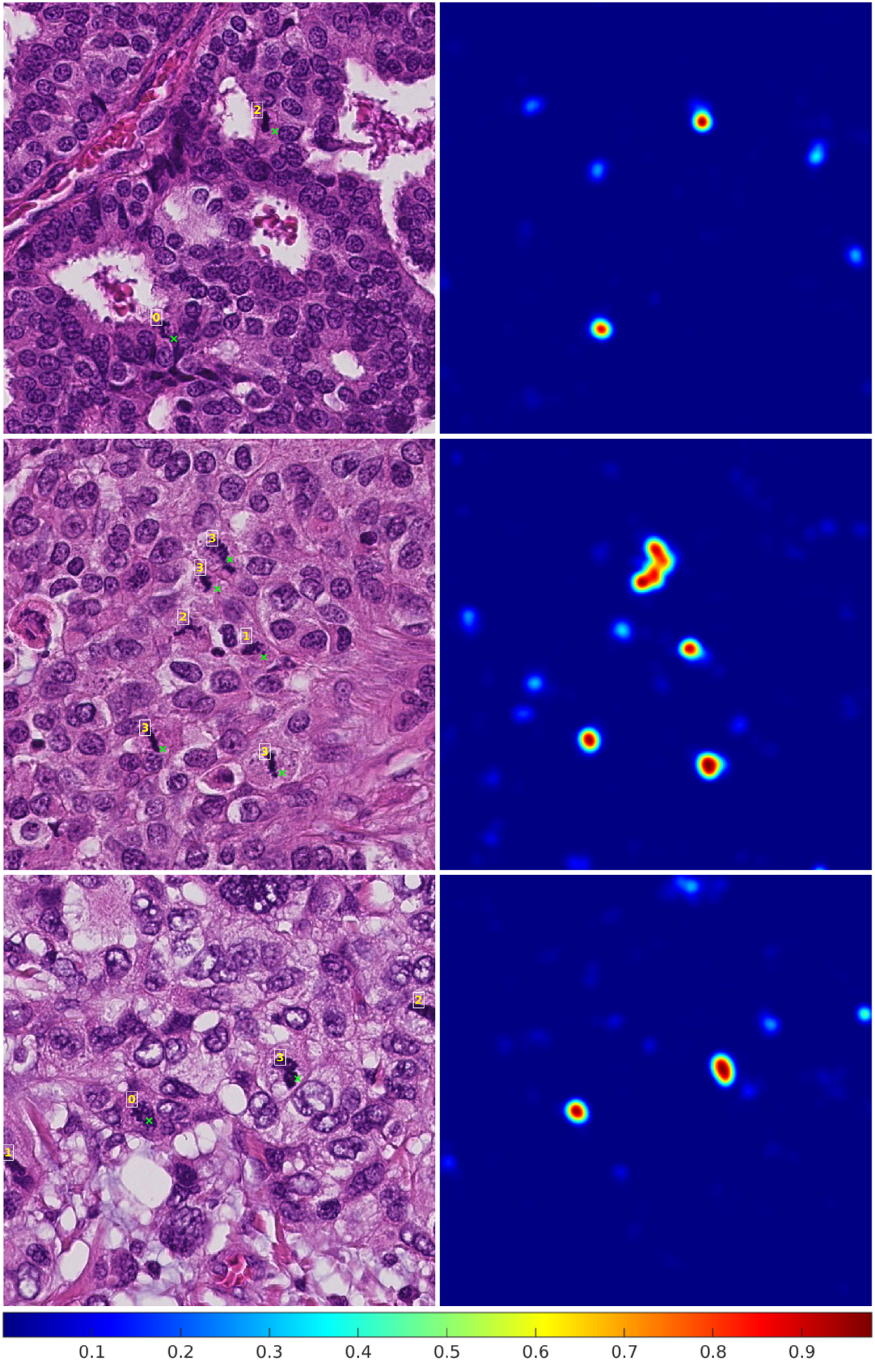
Example results from the automatic mitosis detection algorithm. In the images on the left side, a green cross next to an object indicates a detected mitotic figure and the numbers indicate how many observers annotated the object as a mitotic figure. The corresponding probability maps that are the output of the deep convolutional neural network are given on the right side. All local maxima with a value larger than 0.85 are considered detected mitotic figures.

More details on the training of the deep convolutional neural network model and the design of the automatic mitosis detection method are provided in S1 Appendix. Examples of the automatic mitosis detection are given in Fig 1.

### Measures of observer agreement

The observer agreement for the mitotic count was assessed with the Bland-Altman method.[13] Before calculating the mean difference and limits of agreement, the values were log-transformed because of the right-skewed distribution of the counts. The mitotic score agreement was assessed by analyzing the confusion matrices and computing the quadratic-weighted Cohen's kappa statistic, which puts more weight on cases with larger discrepancy of the score.

The object-level observer agreement was assessed by computing the Dice similarity coefficient.[14] This measure is commonly used in the field of medical image analysis to assess the amount of overlap between two sets of objects. The Dice similarity coefficient for two sets of objects A and B is computed as *D* = 2×|A∩B|/(|A| + |B|). In this expression, |A| and |B| indicate the number of objects in the two sets, and |A∩B| indicates the number of objects that appear in both sets. In the case of mitotic figure counting by two observers, |A| and |B| are the mitotic counts by the two observers and |A∩B| is the number of individual mitotic figures for which they agree. For example, if one observer counted 12 mitotic figures and a second observer counted 14 mitotic figures out of which only 6 were also counted by the first observer, the Dice similarity coefficient would be D = 2×6/(12+14) = 0.46. If the two observers agreed on 10 mitotic figures the value for the Dice similarity coefficient would be 0.76.

The Dice similarity coefficient can take values between 0, meaning that none of the objects appear in both sets, and 1, meaning that the two sets of objects are identical. Additionally, we define the Dice similarity coefficient between two empty sets to be 1. Two annotations or a detection and an annotation are considered to refer to the same object if their distance is less than 30 image pixels or 7.5 μm. This value corresponds approximately to the average size of mitotic figures in the data set, and provides a reasonable tolerance for misalignment of the ground truth location and the detection. The Dice similarity coefficient is computed for both the entire dataset and per individual case.

## Results

### Mitotic count agreement

**Fig 2:**
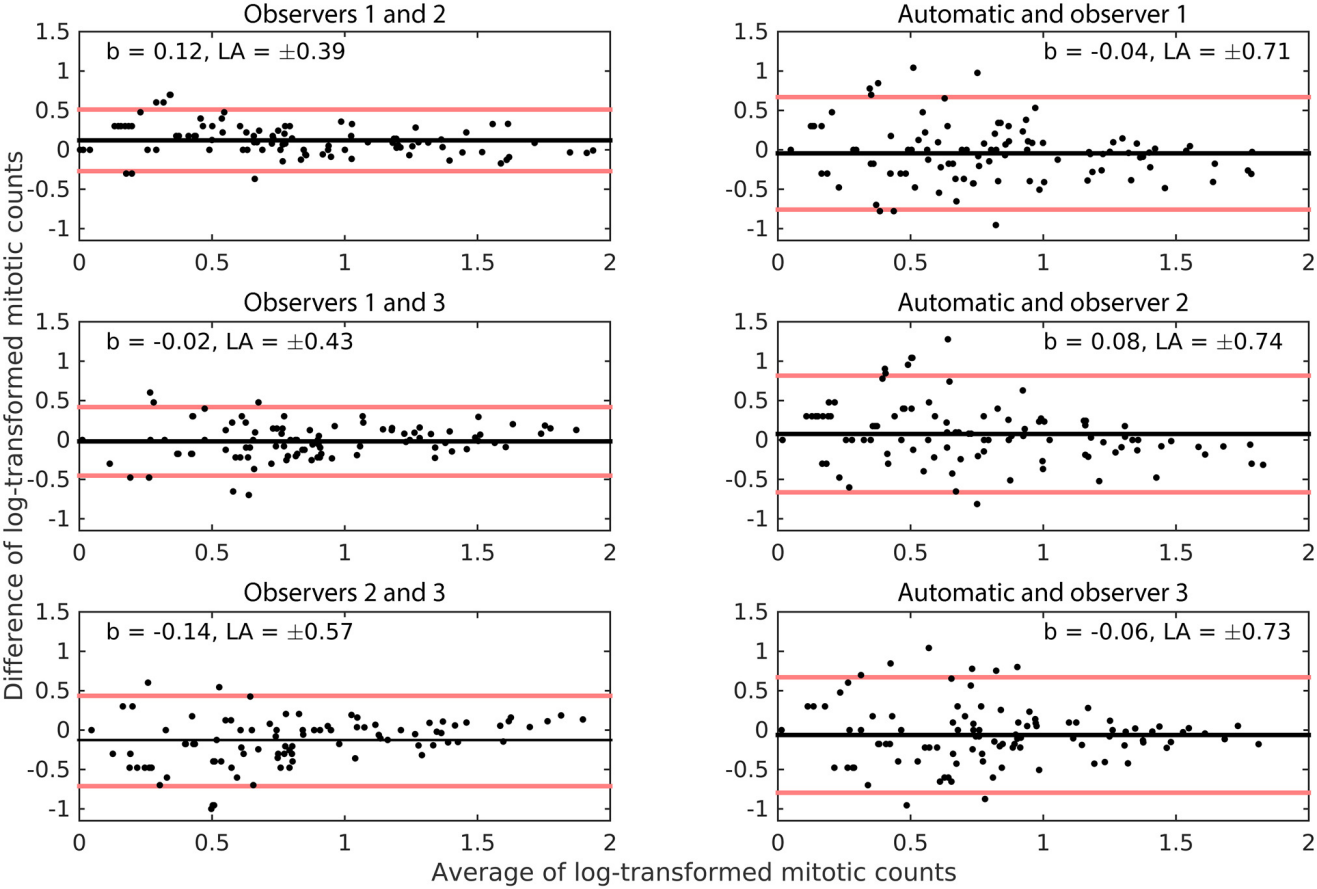
Modified Bland-Altman plots for the log-transformed mitotic counts of the 3 observers and the automatic method. The black line indicates the mean difference *b*, and the red lines indicate the limits of agreement LA. Logarithm with base 10 was used in the transformation. The points are jittered in the horizontal direction to help better visualize overlaps.

The analysis of the Bland-Altman plots, which are given in Fig 2, shows that the mean differences between all pairs of observations were very close to zero. The absolute mean difference was highest between observers 2 and 3 (|*b|* = 0.14). These mean differences are low compared with the range of the log-transformed counts. This means that none of the observers and the automatic method systematically under- or over-estimated the mitotic count to a substantial degree, i.e. there are no biases in the estimation. The limits of agreement indicate that the agreement was better between the three human observers than between the human observers and the automatic method.

### Mitotic score agreement

The results from the analysis of the agreement on the mitotic score between the three observers and the automatic method are shown in Table 1. The quadratic-weighted Cohen's kappa coefficient between the three observers varied from *κ* = 0.7918, CI = [0.6459 0.9378], for observers 2 and 3 to *κ* = 0.8926, CI = [0.7894 0.9957], for observers 1 and 2, which indicates substantial to perfect agreement. For the agreement between the automatic methods and the three observers, this statistic varied between *κ* = 0.6648, CI = [0.4847 0.8448], for observer 3, and *κ* = 0.7416, CI = [0.5805 0.9026], for observer 1. Compared to the inter-observer agreement of the experts this statistic is lower, but nevertheless indicates substantial agreement.

**Table 1:** Confusion matrices for the mitotic activity score for the 3 observers and the automatic method.

	**Score 1**	**Score 2**	**Score 3**	**Score 1**	**Score 2**	**Score 3**	**Score 1**	**Score 2**	**Score 3**
**Score 1**	59	3	0	46	16	0	46	16	1
**Score 2**	3	6	0	3	5	1	3	8	4
**Score 3**	1	6	22	0	5	24	0	2	20
	Observers 1 (rows) vs. 2 (columns)			Observers 1 (rows) vs. 3 (columns)			Observers 2 (rows) vs. 3 (columns)		
	*κ* = 0.8926			*κ* = 0.8324			*κ* = 0.7918		
	CI = [0.7894 0.9957]			CI = [0.7052 0.9596]			CI = [0.6459 0.9378]		
**Score 1**	50	10	2	51	10	2	40	7	2
**Score 2**	5	4	0	7	4	4	17	6	3
**Score 3**	4	3	22	1	3	18	2	4	19
	Observer 1 (rows) vs. automatic (columns)			Observer 2 (rows) vs. automatic (columns)			Observer 3 (rows) vs. automatic (columns)		
	*κ* = 0.7217			*κ* = 0.7416			*κ* = 0.6648		
	CI = [0.5624 0.8809]			CI = [0.5805 0.9026]			CI = [0.4847 0.8448]		

Score 1: 0–6 mitotic figures per 2 mm^2^, score 2: 7–12 mitotic figures per 2 mm^2^, score 3: > 12 mitotic figures per 2 mm^2^. The inter-rater agreement is measured with Cohen's quadratic weighted kappa. CI refers to the 95% confidence interval.

Clinically more relevant are situations in which the disagreement is two score points, i.e. score 1 instead of 3 and vice versa. The analysis of the confusion matrices in Table 1 reveals that there were two such cases of disagreement between the three human observers, and 13 between the human observers and the automatic method.

### Object-level agreement

The Dice similarity coefficients *D* for all pairs of observations for the entire dataset are given in Table 2. For the three observers, the best object-level agreement was achieved between observers 1 and 2 with *D* = 0.6698, CI = [0.6290 0.7106] and the worst between observers 1 and 3 with D = 0.5729, CI = [0.5224 0.6233]. The automatic method had best agreement with observer 2 with *D* = 0.5635, CI = [0.5051 0.6219] and worst with observer 3 with *D* = 0.4993, CI = [0.4415 0.5571].

**Table 2:** Dice similarity coefficients (*D*) for all pairs of observers and the automatic method for the entire dataset.

	**Observer 1**	**Observer 2**	**Observer 3**	**Automatic**
**Observer 1** (*C* = 783)	*D* = 1	*D* = 0.6698	*D* = 0.5729	*D* = 0.5186
		CI = [0.6290 0.7106]	CI = [0.5224 0.6233]	CI = [0.4698 0.5674]
		|A∩B| = 496	|A∩B| = 446	|A∩B| = 369
**Observer 2** (*C* = 698)		*D* = 1	*D* = 0.5883	*D* = 0.5635
			CI = [0.5342 0.6424]	CI = [0.5051 0.6219]
			|A∩B| = 433	|A∩B| = 377
**Observer 3** (*C* = 774)			*D* = 1	*D* = 0.4993
				CI = [0.4415 0.5571]
				|A∩B| = 353
**Automatic** (*C* = 640)				*D* = 1

The analysis is limited to the cases for which ground truth mitotic figures are available from all three observers (*N* = 84). CI refers to the 95% confidence interval computed by bootstrapping. *C* is the number of mitotic figures counted and |A∩B| is the number of objects for which the two observers agreed.

**Fig 3:**
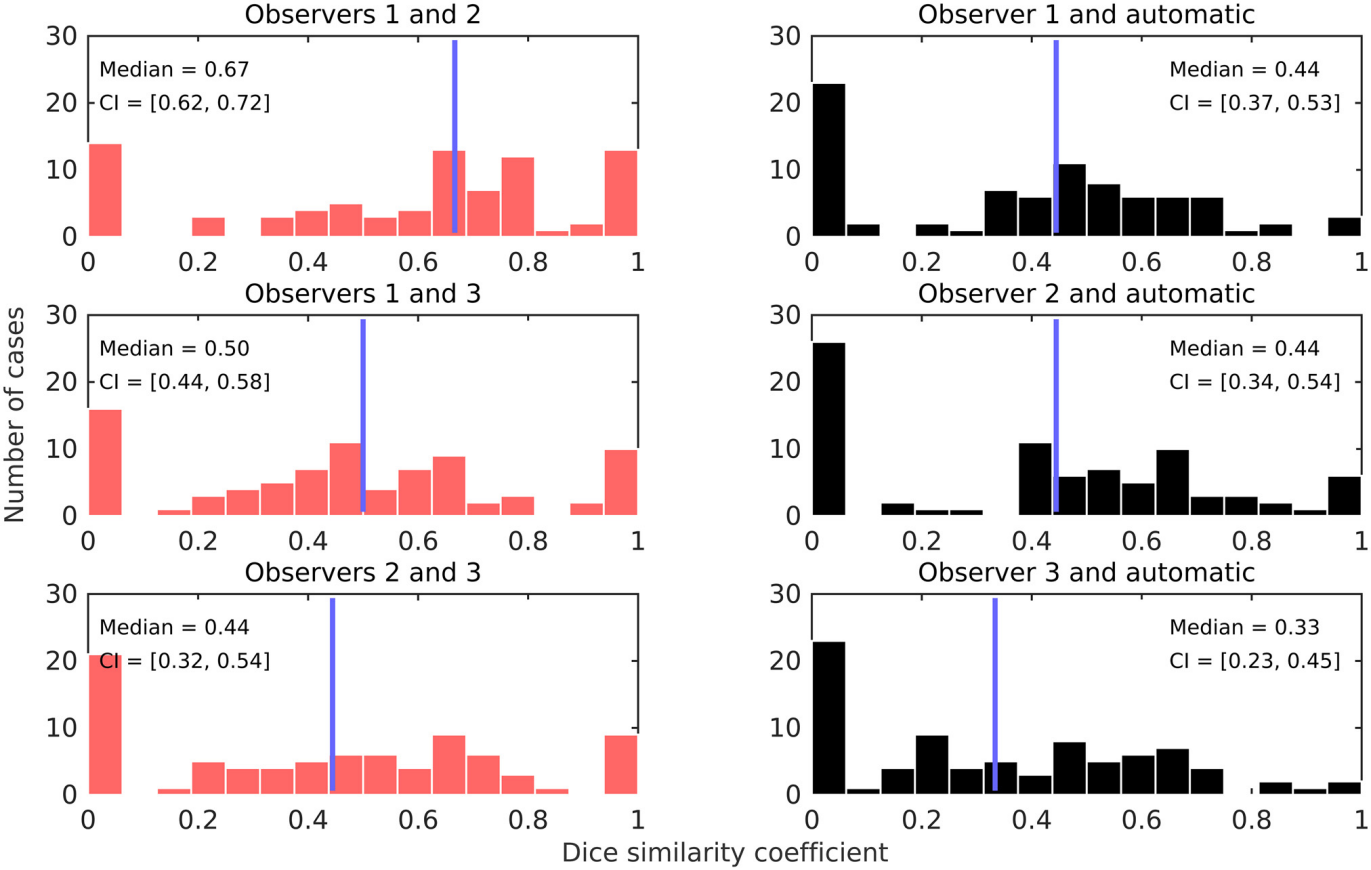
Distribution of the Dice similarity coefficients for individual cases and for all pairs of observations. The blue line indicates the median. The analysis is limited to the cases for which ground truth mitotic figures are available from all three observers (*N* = 84).

Fig 3 shows the Dice similarity coefficients for individual cases and for all pairs of observations. According to these results, the best agreement was achieved between observers 1 and 2 with median Dice similarity coefficient of 0.67, CI = [0.62 0.72]. The agreement was worst between the automatic method and observer 3 with median Dice similarity coefficient of 0.32, CI = [0.22 0.43].

## Discussion and Conclusions

Recent years have brought significant advances in the field of histopathology image analysis, including automatic mitosis detection and counting. State-of-the-art methods are already good candidates for use in clinical practice. However, further methodological advances and validation is needed in order to achieve this goal. The variability of the staining between pathology labs, and even within the same pathology lab at different time points, is a major hurdle in adoption of automatic image analysis methods in the routine workflow, as it directly and adversely affects the performance. With this study, we made one further step towards addressing this issue by training an automatic image analysis method that is robust with respect to staining variability.

One conclusion that can be drawn from the analysis of the results is that the best measured agreement between the pathologists and the automatic method is comparable to the worst measured agreement among the pathologists. This observation is valid across all three types of agreement that were measured. Nevertheless, the performance of the automatic method was on average somewhat worse compared with the three pathologists. It is evident from Table 1 that there are outlier cases where the number of detected mitotic figures by the automatic method was large enough to cause a difference in the mitotic activity score by two points. Although such differences were also present among the pathologists, their number was substantially lower.

When pathologists score a histological slide for mitoses, they first examine the overall appearance of the tissue and then proceed to identifying mitotic figures, i.e. they perform a top-down analysis by including contextual information. The automatic method that was used in this study works in a different way. A decision if a mitotic figure is present at a particular location is made based only on local image data in a 63×63 pixel or 15.75×15.75 μm window around that location. The somewhat worse performance of the automatic method can be in part explained by the lack of inclusion of contextual information. For example, there are instances where the number of mitotic figures is overestimated because of the false detection of hyperchromatic objects that in isolation indeed resemble mitotic figures, but the larger context reveals that they belong to an area of tissue preparation artifacts. The inclusion of contextual information in the detection is the most promising direction for future research.

It should be noted that the mitosis detection model is effectively trained with only 458 mitotic figures from 8 cases, and the remainder of the AMIDA13 dataset was used to monitor for overfitting and determine the optimal operating point of the detector. This number is much smaller than the number of cases and mitotic figures that a typical pathology resident sees during training. Undoubtedly, there are mitotic figure appearance types that are not represented in this dataset, which adversely affected the performance. The expansion of the training dataset is likely to lead to better performance. Furthermore, all three pathologists involved in this study were expert breast cancer pathologists with large experience in the task of mitosis counting. It can be expected that non-expert pathologists would have lower inter-observer agreement that compares more favorably to the agreement with the automatic method.

The addition of a staining normalization procedure and data augmentation by random color and contrast transformation resulted in a method that generalizes well on data from external pathology labs. When both staining normalization, and color and contrast data augmentation were excluded from the training, the resulting method showed good performance on data from the same pathology lab, but the results on the external dataset were notably worse (see the S1 Appendix).

This study compared for the first time the object-level interobserver agreement of mitosis counting among pathologists. Compared with analysis of the mitotic count and the mitotic activity score, an analysis at the level of individual mitotic figures can provide a more in-depth look at the structure of the agreement between the observers.

Analyzing the results from the object-level interobserver agreement in Table 2, it can be concluded that pathologists often do not agree on a particular object. All observers counted a similar number of mitotic figures, however pairwise agreement was achieved only for about two-thirds of the individual objects. The object-level interobserver agreement can be even lower for some individual cases as shown in Fig 3. It should be noted here that almost all of the cases with zero and perfect agreement (Dice similarity coefficients of 0 and 1, respectively), are for cases with only a few mitotic figures, where this is easy to achieve. Such instances disappear when only considering cases with larger number of mitotic figures (see S1 Fig).

**Fig 4:**
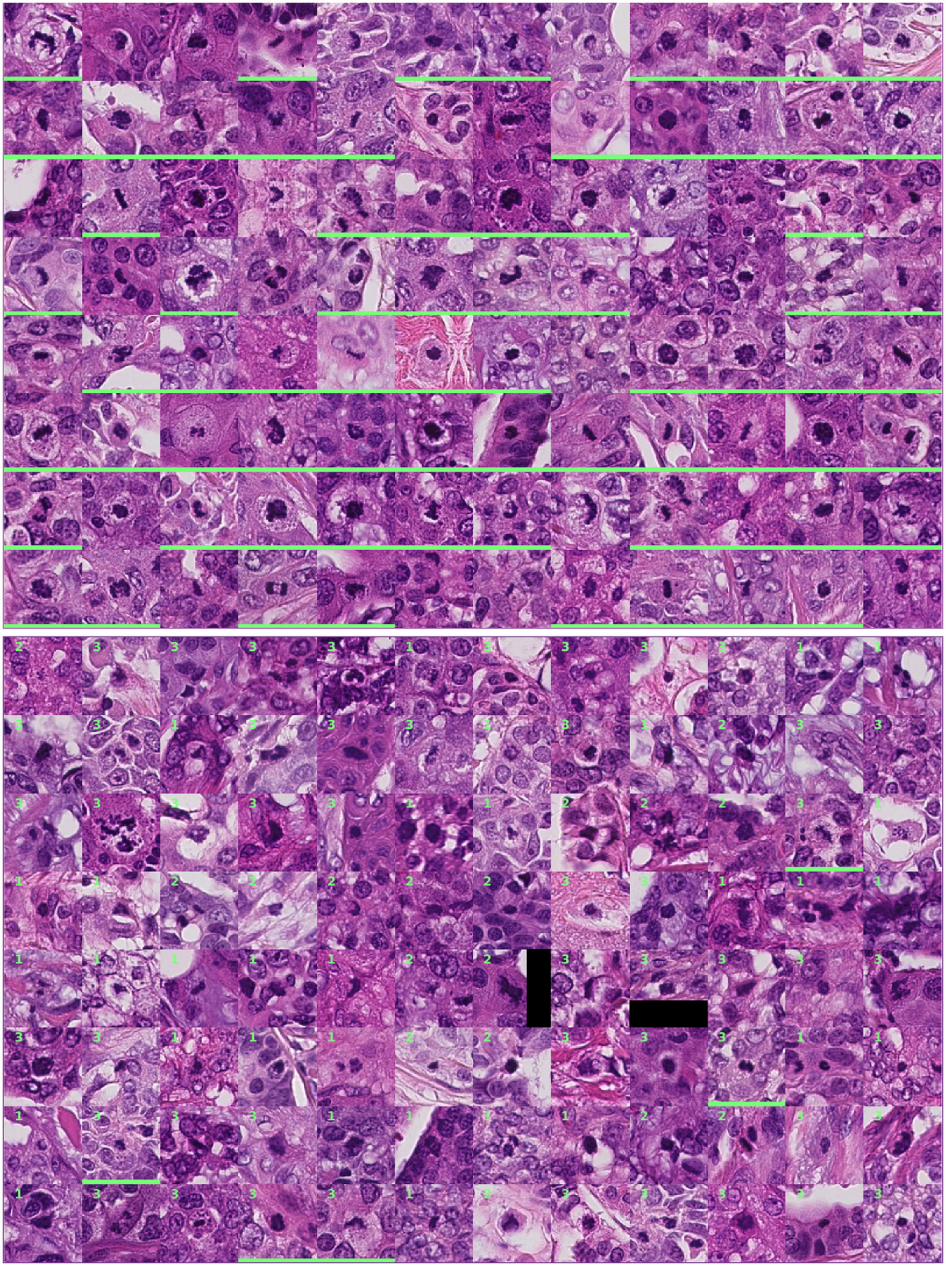
Top (first eight rows): Objects that were annotated as mitotic figures by all three observers. Bottom (last eight rows): Objects that were annotated as mitotic figures by only one observer. The numbers in the top-right corner of the patches indicate the observer that annotated the object. A green bar at the bottom of the image patch indicates that the object was detected by the automatic method.

**Fig 5:**
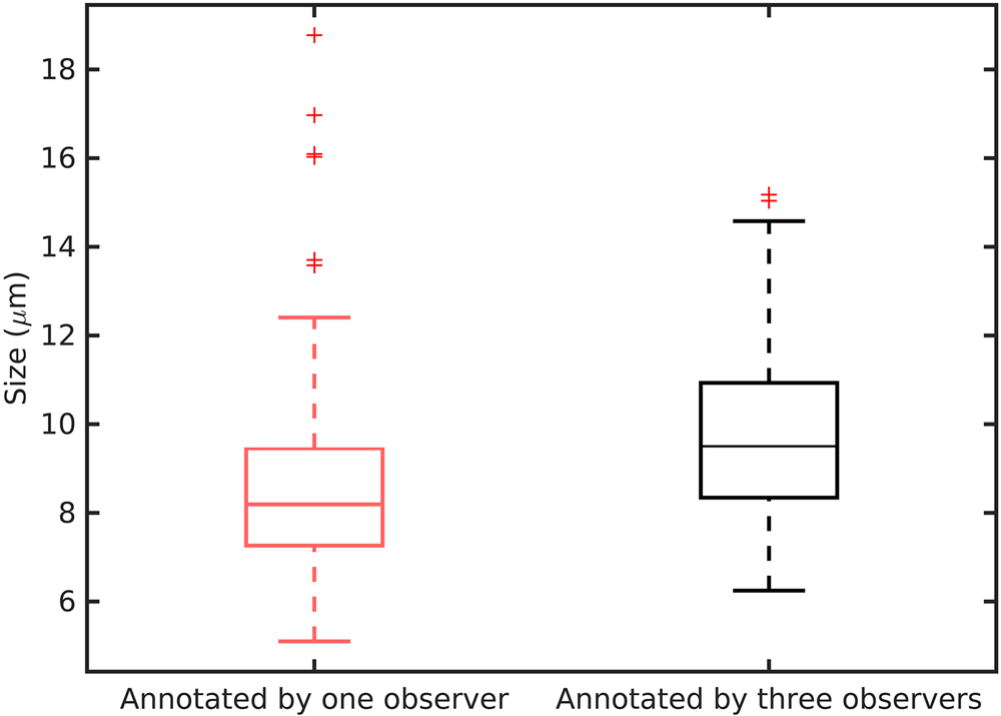
Size of the mitotic figures with poor and good agreement. The mitotic figures annotated by only one observer were on average smaller than those for which all three observers agreed.

Examples of objects that were annotated by all three observers (good agreement) and only by one observer (poor agreement) are shown in Fig 4. It can be argued that some of the objects with low agreement lack one or more of the typical characteristics of mitotic figures. In these instances, subjective decisions were made that were not seconded by another observer. Furthermore, the objects that were annotated by a single observer appear to be on average smaller in size compared with the objects with good agreement. To quantify this observation, we measured the length of the objects at their largest cross-section as an indication of their size (the analysis was limited to the 192 objects in Fig 4). The results from this analysis are illustrated in Fig 5. The average length of the objects with poor agreement was 8.7 μm and for the objects with good agreement 9.8 μm. This difference was statistically significant (*p* < 0.002; two-sample *t*-test). Although the difference in the average sizes is not large, a trend of poor agreement for small objects is evidently present.

The smaller size of the objects with poor agreement is not surprising. These objects are difficult to spot when scanning the slide and identification of the typical appearance characteristics such as the hairy extensions is more difficult due to the small scale at which they appear. The analysis of the object-level agreement suggests that defining a strict size constraint in the mitosis counting protocol might improve the agreement between pathologists.

Fig 5 also reveals that the objects with poor agreement were often not detected by the automatic method. In contrast, a large proportion of the objects with good agreement were also detected by the automatic method.

In conclusion, we describe the results from an object-level interobserver agreement study between three human observers and an automatic method. The automatic method was trained in such a way that it generalizes well to datasets from external pathology labs. The object-level interobserver study revealed that pathologists often do not agree on individual objects. The disagreement is larger for objects from smaller size, which suggests that adding a size constraint in the mitosis counting protocol can improve reproducibility. The automatic mitosis detection method can perform mitosis counting in an unbiased way, with substantial agreement with human experts. Although the agreement between the automatic method and the experts is lower compared to the agreement between the experts, we estimate that this performance is sufficient for use as an interactive tool. In a fully digital pathology workflow, mitosis detection can be performed in the slides before they reach the pathologists. The results from the detection can then be used at the time of diagnosis to guide the pathologists to the region of the tumor with the highest proliferation speed, and present the detected mitotic figures as candidates in order to improve objectivity. In future work, we plan to perform a validation study of such a workflow.

## Supporting Information

S1 Appendix Automatic mitosis detection method description. (DOCX)

S1 Code Implementation of the convolutional neural network in the Caffe deep learning framework. (PROTOTXT)

S1 Fig Similar to Fig 3 in the paper, however the analysis is limited to the cases for which ground truth mitotic figures are available from all three observers and the average mitotic count is larger than 6 (*N* = 30). (TIF)

S2 Fig Example of data augmentation. One training sample is replicated 20 times by employing image transformations. In this way, new and plausible training examples are created. This procedure is called data augmentation.
(TIF)

S3 Fig Similar to Fig 2 in the paper, however this figure shows the Bland-Altman plots for an automatic mitosis detection method without staining normalization during the training and testing. (TIF)

S1 Table Mitotic count and number of mitotic figures with agreement for the three pathologists and the automatic method for individual cases. (XLSX)
