# Supplementary material for: Mitosis Counting in Breast Cancer: Object-Level Interobserver Agreement and Comparison to an Automatic Method
Source: PLoS One. 2016 Aug 16;11(8):e0161286. doi: 10.1371/journal.pone.0161286 (PMC4987048; doi:10.1371/journal.pone.0161286)
Supplement: S1 Appendix — (DOCX) [file pone.0161286.s001.docx]

# Appendix: Automatic mitosis detection method description

The automatic mitosis detection method is based on a convolutional neural network classification model. The inputs to the convolutional neural network are RGB image patches of size 63×63 pixels. For each patch, the neural network outputs a probability estimate that a mitotic figure is present in the center of the patch. When applied to a larger image of a tumor region, a probability value is computed for an image patch centered at every pixel location thus resulting in a probability map (Fig 1). The probability map is spatially smoothed by a disk-shaped averaging filter with radius 10 pixels. Mitotic figures are detected as local maxima above a certain probability threshold in the smoothed probability map. The threshold value (the operating point of the detector) controls the sensitivity of the detector and is subject to optimization.

**Convolutional neural network classification model.** The convolutional neural network consists of four convolutional layers followed by two fully connected layers. The first convolutional layer has a kernel size of 4×4 and the remaining three convolutional layers have a kernel size of 3×3. Each convolutional layer is followed by a max-pooling layer with a kernel size 2×2. All convolutional layers have 16 feature maps. The first fully connected layer has 200 neurons and the second fully connected layer, which is the output layer, has two neurons. ReLU nonlinearities are used throughout the network, except for the output layer that is followed by a softmax nonlinearity that computes the probability output. This architecture is similar to the one used in the winning method of the AMIDA13 challenge. [1,2]

**Training.** The convolutional neural network model was trained with the AMIDA13 training set. [2] Because the AMIDA13 data originates from a single pathology lab it, does not include inter-lab staining variations. In order to improve the generalization of the detector on datasets from other pathology labs, a staining normalization procedure [3,4] was applied to the image data prior to the training of the deep convolutional neural network model. The same procedure is also applied to test images prior to running the detector.

Eight from the 12 cases in the AMIDA13 training set, which contain 458 mitotic figures, were used for training of the model and the remaining four cases were used as a validation set to monitor for over-fitting. Each case in the AMIDA13 dataset is represented with a number of images of size 2000×2000 pixels corresponding to an area of one high power field (HPF). The training and validation samples were formed by extracting 65×65 RGB image patches from the HPFs. The mitosis (positive) class was formed by extracting patches centered at integer locations within 10 pixels around an annotated mitotic figure. The background (negative) class was formed by randomly sampling locations that are at a distance of more than 10 pixels from any annotated mitotic figure.

The training of the convolutional neural network model was performed by batch stochastic gradient descent with momentum (batch size = 64, learning rate = 0.01, momentum = 0.9). During the training, the learning rate was reduced by 10% every 5000 iterations. The weights of the neural network were initialized with small random numbers drawn from a Gaussian distribution, and all biases were initialized to 0.1. The choice for these parameters was based on commonly used values for similar problems in the literature. The training was stopped once the performance on the four validation cases stopped improving. The neural network was implemented and trained with the Caffe [5] deep learning framework (see Code S6 for the implementation).

To avoid over-fitting of the model, which can easily occur because of the number of trainable parameters of the model is much larger than the number of training samples, two regularization strategies were followed. First, L2 regularization (weight decay) of the weights of the neural network was applied during the optimization (weight decay value = 0.0002). Furthermore, the number of training samples was artificially increased by creating new plausible training samples (data augmentation). New training samples were created by employing random transformations that take advantage of the rotational and scaling (for small scaling factors) invariance of the problem. In addition to the random rotation and scaling transformations, small random perturbations of the color and contrast of the patches was performed. These transformations increase the model’s robustness with respect to the staining color and intensity variability, which is not completely eliminated by the staining normalization procedure. An example of data augmentation is given in S2 Fig.

Each positive training sample was replicated 10 times. After extracting the positive samples, an equal number of samples from the background class were extracted. This resulted in approximately 2.7 million samples that were used for training of the model.

With uniform sampling, the majority of the background class will consist of samples that are relatively easy to classify as non-mitosis, such as patches containing only stromal tissue. The number of samples containing “hard” non-mitosis objects, such as lymphocytes and apoptotic nuclei, will be very low. Because these samples will occur less often in the training set, the classification model will not learn to classify them correctly. To remedy this we employed the same boosting-like strategy for “hard” negative samples mining as in Ciresan et al. [1] An initial detector was trained with uniform random sampling of the background class. This detector, which falsely outputs high probabilities for “hard” non-mitosis samples, was then applied to the HPFs in training set. The resulting probability maps were used when extracting the background class for training a new model in such a way that more false positives from the first detector were included. This results in a more challenging training set and leads to significant performance improvement on the AMIDA13 testing set.

**Operating point selection.** The optimal operating point of the detector was selected based on the AMIDA13 testing set. The mitosis detection method was applied to all HPFs in this set. Then, the Dice similarity coefficient was computed for all probability threshold values between 0 and 1 with a step of 0.01. The maximum Dice similarity coefficient *D*_max_ = 0.6572 was obtained for a threshold value of *T* = 0.85. The mitosis detection method with the selected operating point was used when detecting mitotic figures in the independent set of 100 cases used for the observer agreement experiments.

**Influence of staining normalization, and color and contrast augmentation.** To examine the influence of the staining normalization, and the color and contrast data augmentation on the performance of the mitosis detector, two additional experiments were performed.

First, another mitosis detector that does not use staining normalization, and color and contrast augmentation was trained. With the exception of this, the training was performed in the exact same way as the original mitosis detector, including the selection of the optimal operating point. Although the performance of this method on the AMIDA13 testing set was in the same order as the full method (*D*_max_ = 0.6028, *T* = 0.95), the performance on the 100 external cases was notably worse with zero detected mitotic figures for 84 of the cases and 30 detected mitotic figures in total.

In the second experiment, only the staining normalization was omitted. This method also showed good performance on the AMIDA13 testing set (*D*_max_ = 0.6257, *T* = 0.91). The results for the mitotic count agreement are similar to the full method (S3 Fig), however somewhat larger underestimation with respect to observers 1 and 3 is evident. This might be related to poor generalization of the optimal operating point.

The results from these experiments indicate that the use of staining normalization, and color and contrast augmentation results in better performance on external data. They also show that the color and contrast augmentation is a more important factor in achieving this performance improvement.

# References

1. Cireşan DC, Giusti A, Gambardella LM, Schmidhuber J. Mitosis detection in breast cancer histology images with deep neural networks. Medical Image Computing and Computer-Assisted Intervention (MICCAI 2013). 2013. pp. 411–418.

2. Veta M, van Diest PJ, Willems SM, Wang H, Madabhushi A, Cruz-Roa A, et al. Assessment of algorithms for mitosis detection in breast cancer histopathology images. Med Image Anal. 2015;20: 237–248.

3. Macenko M, Niethammer M, Marron JS, Borland D, Woosley JT, Guan X, et al. A method for normalizing histology slides for quantitative analysis. IEEE International Symposium on Biomedical Imaging (ISBI 2009). 2009. pp. 1107–1110.

4. Vink JP, Van Leeuwen MB, Van Deurzen CHM, De Haan G. Efficient nucleus detector in histopathology images. J Microsc. 2013;249: 124–135.

5. Jia Y, Shelhamer E, Donahue J, Karayev S, Long J, Girshick R, et al. Caffe: Convolutional Architecture for Fast Feature Embedding. arXiv:14085093. 2014;
