# Supplementary figures and images for: Mitosis Counting in Breast Cancer: Object-Level Interobserver Agreement and Comparison to an Automatic Method
Source: PLoS One. 2016 Aug 16;11(8):e0161286. doi: 10.1371/journal.pone.0161286 (PMC4987048; doi:10.1371/journal.pone.0161286)

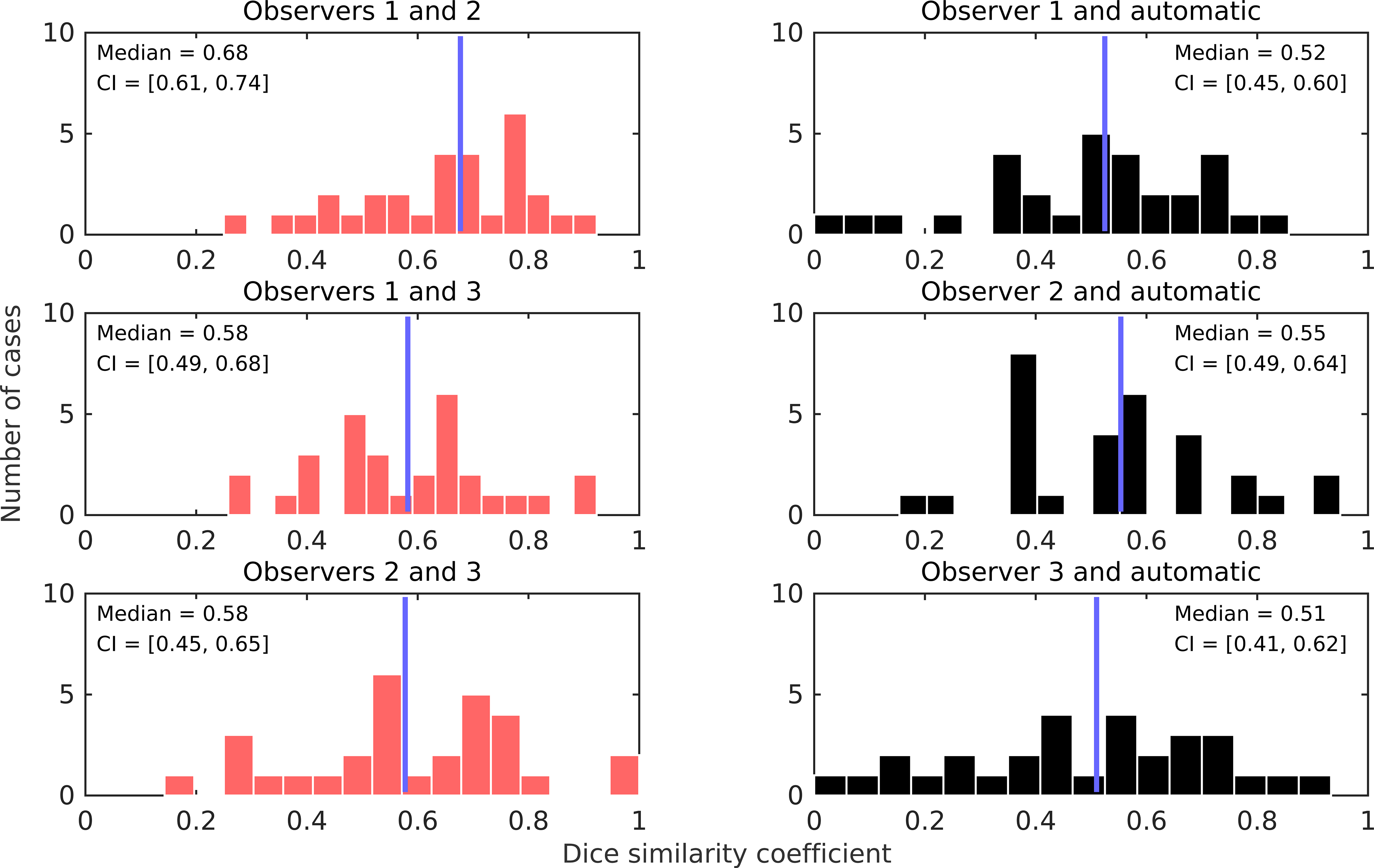

Supplement: S1 Fig — (TIF) [file pone.0161286.s003.tif]

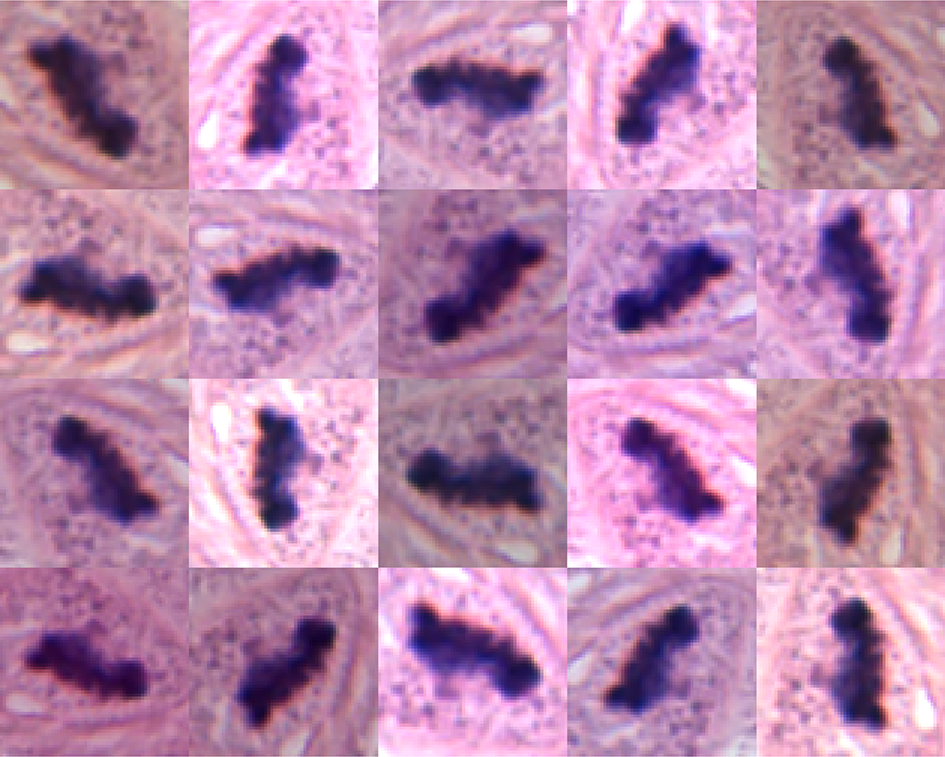

Supplement: S2 Fig — One training sample is replicated 20 times by employing image transformations. In this way, new and plausible training examples are created. This procedure is called data augmentation. (TIF) [file pone.0161286.s004.tif]

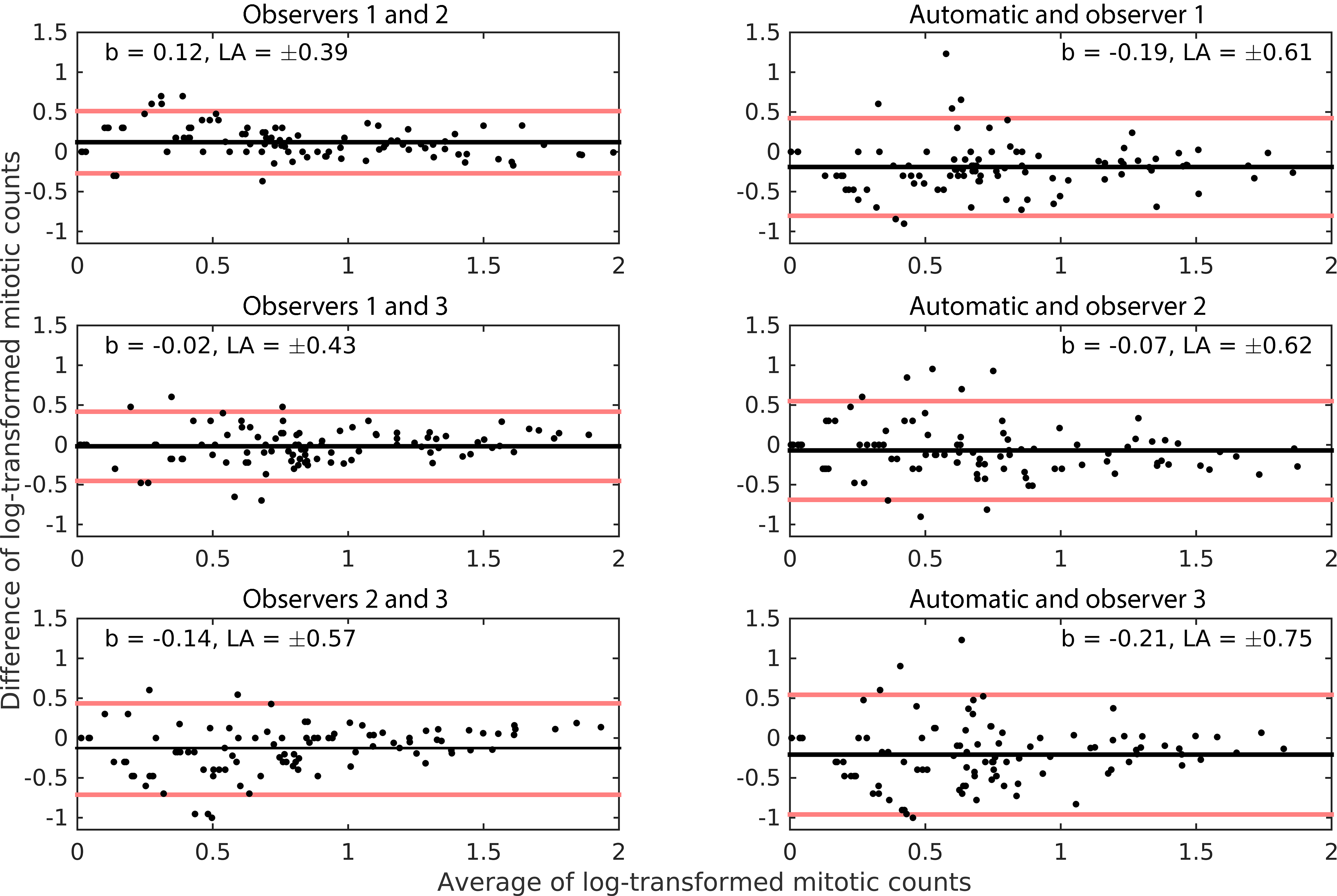

Supplement: S3 Fig — (TIF) [file pone.0161286.s005.tif]
